# Vapour Liquid Solid Growth Effects on InGaN Epilayers Composition Uniformity in Presence of Metal Droplets

**DOI:** 10.3390/nano12213887

**Published:** 2022-11-03

**Authors:** Mani Azadmand, Stefano Vichi, Federico Guido Cesura, Sergio Bietti, Daniel Chrastina, Emiliano Bonera, Giovanni Maria Vanacore, Shiro Tsukamoto, Stefano Sanguinetti

**Affiliations:** 1 Department of Materials Science, University of Milano-Bicocca, 20100 Milano, Italy; 2 INFN, Sezione di Milano-Bicocca, 20100 Milano, Italy; 3 L-NESS, Physics Department, Politecnico di Milano, Via Anzani 42, 22100 Como, Italy

**Keywords:** InGaN, molecular beam epitaxy, metal droplets, vapor–liquid–solid

## Abstract

We investigated the composition uniformity of InGaN epilayers in presence of metal droplets on the surface. We used Plasma Assisted MBE to grow an InGaN sample partially covered by metal droplets and performed structural and compositional analysis. The results showed a marked difference in indium incorporation between the region under the droplets and between them. Based on this observation we proposed a theoretical model able to explain the results by taking into account the vapour liquid solid growth that takes place under the droplet by direct impingement of nitrogen adatoms.

## 1. Introduction

InGaN is a semiconductor with an energy gap that ranges from from 0.7 eV (InN) to 3.4 eV (GaN) depending on its alloy composition [1]. The possibility to cover all the visible spectrum makes InGaN a very interesting material for photovoltaic [2] and optoelectronic [3] applications, such as light emitters and detectors. Moreover, due to its exceptional chemical stability, InGaN can be used for photoelectrochemical applications and biosensing [4,5,6]. Despite the high interest in this material, the growth of InGaN in the entire composition range is still extremely challenging. These difficulties arise from the different thermal stability of In-N and Ga-N bonds and the much larger size of In atoms compared to Ga. The large lattice mismatch of InN and GaN leads to a miscibility gap that can cause fluctuations of the In content in the epilayer [7,8,9]. The calculated effects of strain in the InGaN binodal and spinodal curves show that the miscibility problem remains significant for a large InN mole fraction. InGaN phase separation has also been demonstrated experimentally for both plasma assisted molecular beam epitaxy (PA-MBE) [10] and metalorganic chemical vapor epitaxy (MOCVD) growth [11]. The different In–N and Ga–N bond energies are reflected in the different decomposition temperatures of InN (500–630 ${}^{{}^{\circ}}$C) and GaN (850 ${}^{{}^{\circ}}$C) [12]. Therefore, above 500 ${}^{{}^{\circ}}$C a reduction of In incorporation in the epilayer occurs not only due to the re-evaporation of physisorbed surface adatoms but also due to the thermal decomposition of In-N bonds. Hence, at usual InGaN growth temperatures, namely ≈650 ${}^{{}^{\circ}}$C for MBE and ≈800 ${}^{{}^{\circ}}$C for MOCVD, the incorporation of indium atoms is insufficient to achieve high indium concentrations [7,13,14,15]. One of the possibilities to avoid InGaN decomposition is to grow at low temperature [16]. PA-MBE is the most suitable technique for this purpose since with this equipment the generation of active nitrogen species does not depend on substrate temperature. However, if the growth conditions are not perfectly tuned this could easily lead to a poor crystal quality of the epilayer. This is particularly critical at low growth temperatures since the window for the optimal growth parameters is extremely narrow. If the growth is carried out under metal rich conditions, the excess metal atoms start to accumulate on the surface in form of droplets [17,18,19]. On the other hand, trying to balance this drawback by growing in N-rich conditions will cause 3D growth and a rough surface [19,20,21].

Yamaguchi et al. [22,23] showed that it is possible to grow thick and uniform InGaN films in the entire alloy composition range exploiting the droplet formation and their elimination by radical beam irradiation (DERI). With this method, the Ga and N fluxes are kept constant while the In flux is continuously changed in order to have an alternation of In excess and In interruption, dictated by the necessity to consume the excess of In accumulated on the surface. High In-content InGaN layers were also obtained by using metal-modulated (MME) PA-MBE, growing at low tempeature under constant N flux, while modulating Ga and In fluxes under metal-rich conditions [24,25]. Despite the success of DERI and MME growth techniques, the presence of droplets on the surface and their effect on growth dynamics has been only recently studied [17].

Here we show that the presence of metal droplets on the surface, in addition to the already discussed effect on the growth rate [17], affects the incorporation of In in the InGaN epilayer and causes strong composition fluctuations. We demonstrate that this phenomenon is related to Vapor–Liquid–Solid (VLS) growth that takes place at the interface between the solid and the droplet [26,27].

## 2. Materials and Methods

The sample growth was performed on a single-side polished undoped Si(111) wafer by PA-MBE equipped with a radio frequency (RF) plasma source. As a first step, the native Si oxide was removed in situ by heating the substrate up to 935 ${}^{{}^{\circ}}$C for 10 min. The complete oxide removal was confirmed by RHEED, observing the Si(111) 1 × 1 to 7 × 7 surface reconstruction change. Prior to the InGaN growth, the silicon surface was nitridized by exposing it to an active nitrogen flux of 0.9 sccm with an RF power of 360 W for 5 min at a substrate temperature of 910 ${}^{{}^{\circ}}$C. This resulted in an amorphous SiN${}_{x}$ layer (as confirmed by RHEED), which is known to improve the crystal quality of the epitaxyal InGaN layers grown on top [4,28]. Finally, the InGaN growth was performed at a substrate temperature of 450 ${}^{{}^{\circ}}$C, a nitrogen flux of 0.9 sccm with a RF power of 360 W and Ga and In beam equivalent pressures (BEP) of 5.5 $\times 10^{-8}$ Torr for 90 min. The sample morphology and surface composition were measured by a combination of Scanning Electron Microscopy (SEM) and Energy-Dispersive X-ray Spectroscopy (EDX). SEM-EDX analysis were performed via a FEG-SEM Zeiss Gemini 500 equipped with an in-lens detector for high-resolution imaging and a Bruker QUANTAX X-ray spectrometer. For structural analysis, we carried out X-ray diffraction (XRD) using a PANalytical X’Pert PRO high-resolution diffractometer. The Kα1 radiation from the Cu anode (λ = 0.15406 nm) was selected using a hybrid mirror and 2-bounce Ge monochromator. The sample was mounted on a high-precision goniometer with translational (x, y and z) and rotational (incidence angle ω, diffraction angle 2θ, sample rotation Φ and sample tilt χ) degrees of freedom. A three-bounce Ge monochromator was placed in front of the detector as an analyzer crystal, in order to obtain high precision in 2θ and to reject fluorescence from the sample. ω−2θ scans of the InGaN(0002) peak were obtained, using the Si(111) peak from the substrate as a reference. Finally, to investigate the local variation in the composition [29], line scan Raman spectroscopy was obtained using micro-Raman with an excitation lambda of 532 nm, excitation power of ≈2.5 mW, and spot diameter on the sample of 0.7 μm.

## 3. Results

**Figure 1:**
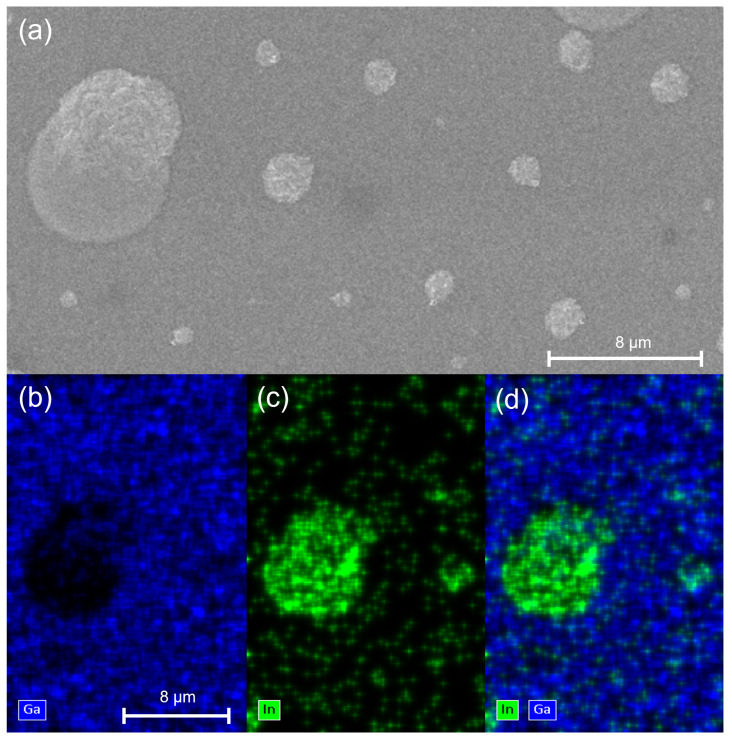
(**a**) SEM image and (**b**–**d**) EDX analysis of a metal droplet. Here are shown separately Ga (**b**) and In (**c**) compositions and their combination (**d**). As can be seen, the metal droplets are almost entirely made of indium.

Figure 1a shows the SEM image of the grown sample. As can be seen, the surface is partially covered by droplets. The droplets exhibit an average size of few μm and are separated by a compact layer. In order to investigate the composition of the metal droplets, EDX was performed and the results are shown in Figure 1b–d. In particular, Figure 1b,c, show the distribution of Ga and In respectively, while Figure 1d combines both of them. It is evident that metal droplets are almost entirely made of In, whereas in the surrounding region both Ga and In are present. Figure 1d clearly shows that in this region the amount of Ga is much larger compared to In, even if the nominal Ga and In fluxes during the growth were equal.

**Figure 2:**
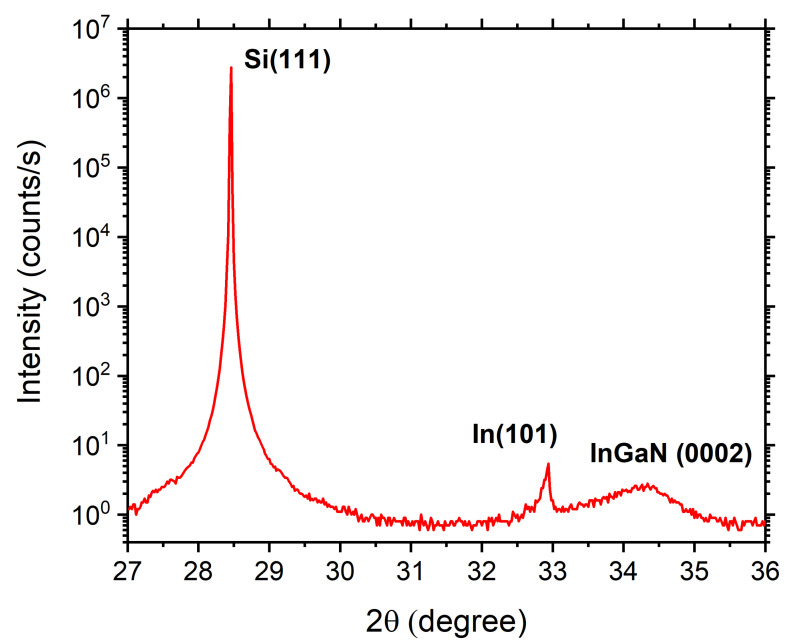
XRD ω−2θ scan in which it is possible to individuate the Si (111) peak at $2\theta =28.5^{{}^{\circ}}$, a sharp In (101) peak at $2\theta =33^{{}^{\circ}}$ due to the diffraction of crystallized indium on the surface and the InGaN (0002) peak at $2\theta =34.3^{{}^{\circ}}$, which corresponds to an In concentration of 13%.

In order to confirm this qualitative observation, we performed XRD ω−2θ scan. The result is shown in Figure 2. The peak at $2\theta =28.5^{{}^{\circ}}$ originates from the Si(111) substrate whereas the sharp peak at $2\theta =33^{{}^{\circ}}$ corresponds to the In (101) diffraction of crystallized In droplets on the surface [30]. The broader peak centered at $2\theta =34.3^{{}^{\circ}}$ is the InGaN(0002) diffraction peak. The In content of the InGaN layer was calculated to be ≈13% by linear interpolation between the lattice constants of InN and GaN. This agrees with the observation based on EDX that the InGaN epilayer was Ga rich.

**Figure 3:**
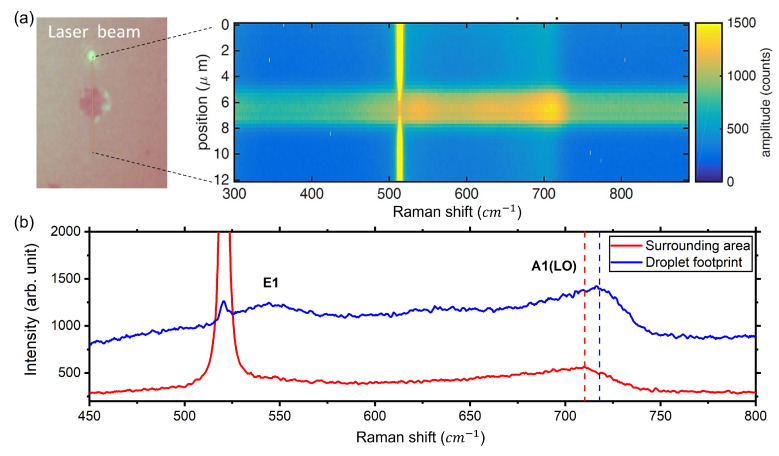
Line scan (**a**) and spot scan (**b**) micro-Raman spectroscopy across the footprint of a metal droplet, revealing the increase in Ga concentration in the droplet footprint with respect to the surroundings. The measured Raman shifts of the A1(LO) peaks are 710 cm${}^{-1}$ (red line) and 718 cm${}^{-1}$ (blue line), corresponding to an indium concentration of 19% and 13% respectively.

In samples grown under metal-rich conditions, it is possible to find droplets’ footprints caused by Ostwald ripening [31], which takes place during the cooling of the substrate. Examples of these footprints can be seen in the SEM image of Figure 1a where they appear as darker spots, while in the EDX analysis they were indistinguishable from the surrounding epilayer. The presence of these features allows for the investigation of the local InGaN composition of the crystallized layer at the bottom of the droplet. Micro-Raman analysis shown in Figure 3a,b revealed a clear increase in Ga concentration in the droplet footprint with respect to the surrounding areas. The Raman line-scan spectrum around the footprint of a metal droplet (Figure 3a) shows a shift in the position of the A1 (LO) peak [32] from 710 cm${}^{-1}$ to 718 cm${}^{-1}$ as the laser beam moves from the area surrounding the droplet to its footprint. The observed Raman shift of the main peak (Figure 3b), highlighted by the dashed lines, corresponds to an In concentration of 13% in the droplet footprint and of 19% in the surrounding area [29]. Tthe spectrum taken in the droplet footprint shows an additional peak at ≈540 cm${}^{-1}$, which could be identified as the E2 mode [33], and a second one less intense at ≈630 cm${}^{-1}$, which is typically addressed to compositional inhomogeneities [33].

These results clearly indicate a complex growth dynamic in presence of droplets when ternary compounds are involved. In particular, we have observed a strong inhomogeneity in the In incorporation between and under the droplets.

## 4. Discussion

In order to explain the experimental observations, we propose here a theoretical model that describes the growth dynamic under the droplets. This model is able to explain the composition of the metal droplets and of the epilayer below when ternary III/V are grown. As soon as droplets begin to form on the surface, a new growth channel starts due to the VLS process that takes place under the droplets [17]. In the most general case, when a droplet composed of two metals is irradiated with a flux of a group V element, the VLS growth mode takes place at the liquid–solid interface. This process leads to the segregation of the metal with the higher reactivity with the group V element.

As a matter of fact, the VLS growth at the interface involves two crystallization reactions of the two metal species (Ga and In) and the group V element (N) in the liquid: $Ga^{l}+N^{l}\to GaN^{s}$ and $In^{l}+N^{l}\to InN^{s}$. If the activity coefficients are independent of the concentrations, we can write the ratio of the law of mass action as [34]:

(1) $$\frac{u_{In}}{u_{Ga}}\frac{x_{GaN}}{x_{InN}}=\frac{Q_{GaN}}{Q_{InN}}=\epsilon$$

Here $u_{Ga}$ and $u_{In}$ are the Ga and In mole fractions in the droplet, $x_{GaN}=\zeta_{Ga}$ and $x_{InN}=1-\zeta_{Ga}$ are the InN and GaN mole fractions in the $In_{1-\zeta }Ga_{\zeta }N$ growing layer. $Q_{GaN}$ and $Q_{InN}$ are the reaction quotients (which at equilibrium become the equilibrium constants $K_{GaN}$ and $K_{InN}$ ), respectively. In case of Ga and In, since the enthalpy of formation of GaN ($\Delta H_{GaN}=157\pm 16$ kJ/mol) is much larger than the one of InN ($\Delta H_{InN}=29\pm 9$ kJ/mol ), the reaction quotient ratio is ϵ≫1.

Therefore, the compositions of the more reactive element (in our case Ga) in the liquid droplet ($u_{Ga}$) and in the solid ($\zeta_{Ga}$) are related by [34]:

(2) $$\zeta_{Ga}\left(u_{Ga}\right)=\frac{u_{Ga}\epsilon }{1+\left(\epsilon -1\right)u_{Ga}}$$

On the basis of Equation (2), being ϵ≫1, we expect a strong increase of the Ga concentration $\zeta_{Ga}$ in the layer growing under the droplet with respect to the concentration α calculated based on Ga and In fluxes. The outcome of this process is that the excess of Ga, with respect to steady state conditions within the droplet, will be segregated at the interface, leaving an excess of In in the droplet. The rate equation governing the molar fraction evolution with the deposition time of Ga in the droplet, $u_{Ga}$, and in the bulk, $\zeta_{Ga}$ is [34]:

(3) $$\frac{du_{Ga}}{dt}=-G\zeta_{Ga}\left(u_{Ga}\right)+K$$

where we have considered that mole fraction $u_{Ga}$ depends on two contributions: (1) the loss of atoms due to the incorporation in the crystal under the droplet $-G\zeta_{Ga}\left(u_{Ga}\right)$, where *G* is the adatom incorporation rate in the crystal by VLS, which depends on the Nitrogen flux incorporated in the droplet, and (2) the flux of Ga atoms impinging on the droplet at constant flux from the growth environment, normalized to the total number of atoms present in the droplet, *K*. Under droplet growing conditions, that is whenever the flux of each metal element is larger than the incorporation rate at the bottom of the droplet (i.e., $K-G\zeta_{Ga}\left(u_{Ga}\right)\gg 0$), the solution of Equation (3), for ϵ≫1, is

(4) $$u_{Ga}\gg \frac{K}{\epsilon (G-K)}.$$

Combining Equations (2) and (4), the Ga concentration $\zeta_{Ga}$ in the InGaN layer growing by VLS under the droplet is $\zeta_{Ga}\gg \frac{K\epsilon }{G\epsilon -K}$ which, being ϵ≫1, reduces to:

(5) $$\zeta_{Ga}\gg \frac{K}{G}.$$

Under droplet growing conditions, K>αG so that the concentration of Ga under the droplet is $\zeta_{Ga}\gg \alpha$. As a consequence, the VLS growth under the droplets strongly favors the Ga segregation at the droplet footprint, which due to the large value of ϵ can be considered close to pure GaN.

These predictions agree well with the experimental results shown in Figure 1 and Figure 3. The application of the model to our sample results in two predictions: the metal droplets are mostly made of indium and the InGaN layer grown below the droplets is more Ga-rich than the one grown in between droplets. Both these predictions are in agreement with the observations of Figure 1 and Figure 2 respectively.

## 5. Conclusions

In this work we have shown that when growing ternary compounds the presence of metal droplets on the surface has a detrimental effect on the composition uniformity of the epilayer. We addressed this effect to the VLS growth mode favoring the Ga segregation at the interface between the droplets and the substrate and we discussed a theoretical model which describes the growth dynamics under these circumstances. These findings suggest that even if the growth modes which take advantage of droplet formation (in particular MME [35,36] and DERI [22,23]) work nicely with binary compounds, they may lead to local composition fluctuations if applied to grow ternary compounds.

## Data Availability

Not applicable.

## References

[1] Yam F.K., Hassan Z. (2008). InGaN: An overview of the growth kinetics, physical properties and emission mechanisms. Superlattices Microstruct..

[2] Hsu L., Walukiewicz W. (2008). Modeling of InGaN/Si tandem solar cells. J. Appl. Phys..

[3] Tchernycheva M., Messanvi A., De Luna Bugallo A., Jacopin G., Lavenus P., Rigutti L., Zhang H., Halioua Y., Julien F.H., Eymery J. (2014). Integrated photonic platform based on InGaN/GaN nanowire emitters and detectors. Nano Lett..

[4] Aseev P., Rodriguez P.E.D.S., Gómez V.J., Alvi N.U.H., Mánuel J.M., Morales F.M., Jiménez J.J., García R., Senichev A., Lienau C. (2015). Near-infrared emitting In-rich InGaN layers grown directly on Si: Towards the whole composition range. Appl. Phys. Lett..

[5] Alvi N.H., Soto Rodriguez P.E., Gómez V.J., Kumar P., Amin G., Nur O., Willander M., Nötzel R. (2012). Highly efficient potentiometric glucose biosensor based on functionalized InN quantum dots. Appl. Phys. Lett..

[6] Alvi N.U.H., Rodriguez P.E., Gómez V.J., Kumar P., Willander M., Nötzel R. (2013). Highly sensitive and fast anion-selective InN quantum dot electrochemical sensors. Appl. Phys. Express.

[7] Iliopoulos E., Georgakilas A., Dimakis E., Adikimenakis A., Tsagaraki K., Androulidaki M., Pelekanos N.T. (2006). InGaN(0001) alloys grown in the entire composition range by plasma assisted molecular beam epitaxy. Phys. Status Solidi (A) Appl. Mater. Sci..

[8] Ho I.H., Stringfellow G.B. (1996). Solid phase immiscibility in GaInN. Appl. Phys. Lett..

[9] Wakahara A., Tokuda T., Dang X.Z., Noda S., Sasaki A. (1997). Compositional inhomogeneity and immiscibility of a GaInN ternary alloy. Appl. Phys. Lett..

[10] Doppalapudi D., Basu S.N., Ludwig K.F., Moustakas T.D. (1998). Phase separation and ordering in InGaN alloys grown by molecular beam epitaxy. J. Appl. Phys..

[11] El-Masry N.A., Piner E.L., Liu S.X., Bedair S.M. (1998). Phase separation in InGaN grown by metalorganic chemical vapor deposition. Appl. Phys. Lett..

[12] Ambacher O., Brandt M.S., Dimitrov R., Metzger T., Stutzmann M., Fischer R.A., Miehr A., Bergmaier A., Dollinger G. (1996). Thermal stability and desorption of group III nitrides prepared by metal organic chemical vapor deposition. J. Vac. Sci. Technol. Microelectron. Nanometer Struct..

[13] Dimakis E., Georgakilas A., Androulidaki M., Tsagaraki K., Kittler G., Kalaitzakis F., Cengher D., Bellet-Amalric E., Jalabert D., Pelekanos N.T. (2003). Plasma-assisted MBE growth of quaternary InAlGaN quantum well heterostructures with room temperature luminescence. J. Cryst. Growth.

[14] Böttcher T., Einfeldt S., Kirchner V., Figge S., Heinke H., Hommel D., Selke H., Ryder P.L. (1998). Incorporation of indium during molecular beam epitaxy of InGaN. Appl. Phys. Lett..

[15] Bord O.V., Talalaev R.A., Karpov S.Y., Makarov Y.N. (1999). Indium incorporation and droplet formation during InGaN molecular beam epitaxy. Phys. Status Solidi (A) Appl. Res..

[16] Kumar P., Rodriguez P.E., Gómez V.J., Alvi N.H., Calleja E., Nötzel R. (2013). First demonstration of direct growth of planar high-in-composition InGaN layers on Si. Appl. Phys. Express.

[17] Azadmand M., Barabani L., Bietti S., Chrastina D., Bonera E., Acciarri M., Fedorov A., Tsukamoto S., Nötzel R., Sanguinetti S. (2018). Droplet Controlled Growth Dynamics in Molecular Beam Epitaxy of Nitride Semiconductors. Sci. Rep..

[18] Heying B., Averbeck R., Chen L.F., Haus E., Riechert H., Speck J.S. (2000). Control of GaN surface morphologies using plasma-assisted molecular beam epitaxy. J. Appl. Phys..

[19] Adelmann C., Brault J., Jalabert D., Gentile P., Mariette H., Mula G., Daudin B. (2002). Dynamically stable gallium surface coverages during plasma-assisted molecular-beam epitaxy of (0001) GaN. J. Appl. Phys..

[20] Tarsa E.J., Heying B., Wu X.H., Fini P., DenBaars S.P., Speck J.S. (1997). Homoepitaxial growth of GaN under Ga-stable and N-stable conditions by plasma-assisted molecular beam epitaxy. J. Appl. Phys..

[21] Fernández-Garrido S., Grandal J., Calleja E., Sánchez-García M.A., López-Romero D. (2009). A growth diagram for plasma-assisted molecular beam epitaxy of GaN nanocolumns on Si(111). J. Appl. Phys..

[22] Yamaguchi T., Uematsu N., Araki T., Honda T., Yoon E., Nanishi Y. (2013). Growth of thick InGaN films with entire alloy composition using droplet elimination by radical-beam irradiation. J. Cryst. Growth.

[23] Yamaguchi T., Nanishi Y. (2009). Indium droplet elimination by radical beam irradiation for reproducible and high-quality growth of InN by RF molecular beam epitaxy. Appl. Phys. Express.

[24] Moseley M., Lowder J., Billingsley D., Doolittle W.A. (2010). Control of surface adatom kinetics for the growth of high-indium content InGaN throughout the miscibility gap. Appl. Phys. Lett..

[25] Moseley M., Gunning B., Greenlee J., Lowder J., Namkoong G., Alan Doolittle W. (2012). Observation and control of the surface kinetics of InGaN for the elimination of phase separation. J. Appl. Phys..

[26] Yin Y., Sun H., Sang L., Chen P., Zheng Y., Dierre B., Sumiya M., Shi Y., Sekiguchi T. (2015). Influence of dislocations on indium diffusion in semi-polar InGaN/GaN heterostructures. AIP Adv..

[27] Fang H., Yang Z.J., Wang Y., Dai T., Sang L.W., Zhao L.B., Yu T.J., Zhang G.Y. (2008). Analysis of mass transport mechanism in InGaN epitaxy on ridge shaped selective area growth GaN by metal organic chemical vapor deposition. J. Appl. Phys..

[28] Nakada Y., Aksenov I., Okumura H. (1998). GaN heteroepitaxial growth on silicon nitride buffer layers formed on Si (111) surfaces by plasma-assisted molecular beam epitaxy. Appl. Phys. Lett..

[29] Azadmand M., Bonera E., Chrastina D., Bietti S., Tsukamoto S., Nötzel R., Sanguinetti S. (2019). Raman spectroscopy of epitaxial InGaN/Si in the central composition range. Jpn. J. Appl. Phys..

[30] Lu H., Thothathiri M., Wu Z., Bhat I. (1997). Study of indium droplets formation on the InxGa1-xN films by single crystal X-ray diffraction. J. Electron. Mater..

[31] Voorhees P.W. (1985). The Theory of Ostwald Ripening. J. Stat. Phys..

[32] Grille H., Schnittler C., Bechstedt F. (2000). Phonons in ternary group-III nitride alloys. Phys. Rev.-Condens. Matter Mater. Phys..

[33] Alexson D., Bergman L., Nemanich R.J., Dutta M., Stroscio M.A., Parker C.A., Bedair S.M., El-Masry N.A., Adar F. (2001). Ultraviolet Raman study of A1(LO) and E2 phonons in InxGa1-xN alloys. J. Appl. Phys..

[34] Priante G., Glas F., Patriarche G., Pantzas K., Oehler F., Harmand J.C. (2016). Sharpening the Interfaces of Axial Heterostructures in Self-Catalyzed AlGaAs Nanowires: Experiment and Theory. Nano Lett..

[35] Namkoong G., Trybus E., Lee K.K., Moseley M., Doolittle W.A., Look D.C. (2008). Metal modulation epitaxy growth for extremely high hole concentrations above 1019 cm-3 in GaN. Appl. Phys. Lett..

[36] Clinton E.A., Vadiee E., Fabien C.A., Moseley M.W., Gunning B.P., Doolittle W.A., Fischer A.M., Wei Y.O., Xie H., Ponce F.A. (2017). A review of the synthesis of reduced defect density InxGa1−xN for all indium compositions. Solid-State Electron..

